# Subjective Health Status, Health-Related Behavior, and Health Literacy of Health Professional Students: Results from a Cross-Sectional Study

**DOI:** 10.3390/healthcare12020277

**Published:** 2024-01-22

**Authors:** Ivonne-Nadine Jürgensen, Peter Koch, Ramona Otto, Annike Morgane Nock, Corinna Petersen-Ewert

**Affiliations:** 1 Department of Nursing and Management, Faculty of Business and Social Science, University of Applied Sciences Hamburg, 20099 Hamburg, Germany; annike.nock@haw-hamburg.de (A.M.N.); corinna.petersen-ewert@haw-hamburg.de (C.P.-E.); 2 Competence Center for Epidemiology and Health Services Research for Healthcare Professionals (CVcare), Institute for Health Services Research in Dermatology and Nursing (IVDP), University Medical Center Hamburg-Eppendorf, 20246 Hamburg, Germany; p.koch@uke.de (P.K.); r.otto@uke.de (R.O.)

**Keywords:** health status, health behavior, health literacy, health professional students, Germany, study

## Abstract

The importance of health promotion for health professional students is increasingly crucial, as the rising requirements for those students can have a negative impact on their health. Despite this awareness, there is still limited knowledge in Germany about the specific health needs of this group of students. This study’s aim was, therefore, to assess the subjective health of first-year students and to identify health needs. Thus, this study is based on an online survey using standardized measurement instruments. First-year students from three degree programs were included. The data were analyzed descriptively. A total of *n* = 98 (72.6%) participated in the survey. The results showed that a major proportion of participants (80.3%) rated their health positively, but a significant proportion reported weight disorders (24%) and pre-existing health conditions (62.1%) at baseline. Interestingly, a high proportion (59%) reported a high level of mental well-being at the time of the survey. However, worrying findings regarding lifestyle behaviors, including physical inactivity (40.6%), smoking (20%), risky alcohol use (24%), and unhealthy eating habits (37%) were determined. In terms of health literacy, around 45% of students rated their health competencies as problematic. Furthermore, it was found that students with low health literacy had a significantly higher prevalence of low mental well-being (53.3% vs. 30.8%, *p* = 0.036) and unhealthy eating habits (48.8% vs. 26.5%, *p* = 0.027) compared to students with sufficient health literacy. Health professional students should be considered a relevant target group for health and health competence promotion from the beginning of their studies. The identified fields of action should be addressed in the context of health promotion. This is of particular importance as they are not only aimed at improving the students’ well-being but also will later work directly with patients and, therefore, have a direct influence on the health of others.

## 1. Introduction

Background. Starting a university degree program is a unique phase of life that presents numerous challenges for both young adults [1,2] and for already experienced health professionals who choose to study [3]. This stage of life can particularly be challenging for young adults due to the confrontation of several new demands. These demands include factors such as navigating the university setting, making new contacts, and probably adjusting to a new city [2,4]. Moreover, health professional students in Germany especially face the challenge of managing academic and clinical demands [3,5]. Coping with these demands may lead students to disregard their own health undesirably [6,7].

Consequently, health promotion initiatives in higher education settings are crucial to promoting students’ overall health and well-being [8,9]. Leading the Systemic requirements-resources model in health promotion. Therefore, a comprehensive view of health is helpful in the context of health promotion at universities and should be integrated into a theoretical model. This allows different conceptual and methodological approaches to be based on and integrated as a part of this framework [10]. The Systemic requirements-resources model (SAR), developed by Becker [11], provides such a framework. It views health and disease as the result of adaptation and regulation processes between an individual and their external environment. In the context of the SAR model, health promotion can be characterized as the improvement of the conditions for coping with external and internal demands through external and internal resources [10,11]. This raises the question for us of how first-year students assess their health, health behavior, and competencies. The aim of this survey is, therefore, to provide comprehensive insight into the health situation of this specific group of students and address a knowledge gap in Germany [12]. Shifting to the concept of health. The World Health Organization (WHO) defines health as a state of complete physical, mental, and social well-being that goes beyond the mere absence of disease or infirmity [13]. This definition highlights the holistic perspective of health, considering it as a resource for daily living, rather than a singular life goal [8]. As a result, health encompasses a positive concept that includes social and personal resources as well as physical capabilities [13]. Based on the broad understanding of the WHO, it is crucial in the context of health promotion to understand this complex dimension of health scientifically [14]. The scientific study of health can be both objective (e.g., through medical examinations) and subjective (e.g., asking people about their subjective health) [13]. Subjective health is of particular importance in the context of prevention and health promotion, as it considers the whole life situation of the individual and not only the bio-medical aspects [15]. Scientific assessments of health status often involve standardized questionnaires that capture self-perceived health status and health-related behavior, thus accounting for the subjective facet of health [16]. Transition to the health knowledge of health professionals in Germany. Health-related occupations are associated with demanding work environments. Among these, the nursing profession is particularly associated with constant high physical and mental demands, e.g., shift work, time pressure, and emotional stress [17,18]. Women in this occupational field also have the challenge of balancing work and family life, which is particularly important due to the high proportion of women in this sector [17]. As a result, consequences include a low job retention rate, increasing absenteeism, and significant turnover [17,19]. Several primary research studies [20,21,22,23] and secondary data studies [18] along with health insurance data in Germany [23,24] have therefore moved the health and working conditions of health professionals into the focus of scientific consideration to assess and describe their health and determinants. Data from health insurance indicate rising absenteeism due to musculoskeletal disorders and mental health issues [23,25], as well as respiratory diseases [19]. Consequently, occupational health promotion emerges as an important strategy to meet health demands and promote work capacity [17,18]. In summary, substantial evidence exists regarding the health situation of this specific occupational group in Germany. However, a recent scoping review by the authors shows only limited evidence on the health situation of health professional students [12]. This limitation makes it difficult to consider and support distinct health needs and academic challenges in a university setting. Crossover to health promotion in the university setting. It is important to recognize that health promotion is a universal value that is supported by government institutions. Health promotion empowers individuals to enhance their health by gaining more control over their health determinants [13]. In line with this perspective, an international expert group outlined the Okanagan Charter in 2015, spotting the university as an essential setting for health promotion. Importantly, students, who are the future professionals and leaders of the world, constitute a target group for health promotion interventions [26].

Moreover, a German law underlines this position through the Prevention Act (2015) and emphasizes the importance of supporting health and health literacy for each student [27]. Health promotion at the Department of Nursing and Management (University of Applied Sciences Hamburg). The Department of Nursing and Management (P&M) cares about the health of its students. The head of the department is aware that studying health professions is a challenge and places high demands and responsibility on students. The aim is to enable students to study healthily and to strengthen their subjective health and resources. Therefore, the study aims to assess health variables of first-year students in three selected degree programs who have not yet been comprehensively surveyed regarding their health.

Objectives. This study consists of three aims and research questions. The aims are (i) to describe the subjective health status, health-related behaviors, and health literacy of first-year health professional students; (ii) to examine the differences between the students and examine health outcomes in relation to health literacy level; (iii) to point out students’ health needs based on the findings. The research aims to answer the following research questions:

1. How do health professional students assess their health status, health-related behavior, and health literacy level at the beginning of their studies?
2. Are there differences between students in terms of their health status, health-related behavior, and health literacy level?
3. Are there differences in the prevalence of health and health-related behavior between students with sufficient and low health literacy?

## 2. Materials and Methods

### 2.1. Study Design

Descriptive Study. The research was based on a cross-sectional survey. Due to COVID-19 restrictions, the survey was conducted online. Lime Survey was selected as the software for the online survey. Lime Survey offers a flexible questionnaire design, a user-friendly interface, secure data management, and enables the formulation of information and consent forms (Lime Survey: https://www.limesurvey.org/de (accessed on 1 November 2023). The data collection took place from April 2021 to June 2021 and from April 2022 to July 2022. Students were given access to the survey link, which was provided by the author (INJ) and the lecturers both by email and during the online lessons via MS Teams. They were informed about the aim and purpose of voluntary participation in the survey. To participate in the survey, students were asked to click on the link provided. All responses were anonymized, and the data were used for scientific purposes only. To ensure that participants understood the purpose of the survey and the terms and conditions, the explicit consent box in the survey tool had to be ticked before accessing the online questionnaire. This box served as written consent to participate and made it clear that the participants had understood the privacy policy and the purpose of the survey. The survey was announced with an estimated time frame of 30 min. Participants were able to submit their responses electronically by clicking “Submit”. The collected data were saved in the survey tool and transferred to SPSS for statistical analysis. The survey was approved by the CCG Ethics Committee of the Hamburg University of Applied Sciences.

### 2.2. Study Setting and Survey Participants

Study setting. The study setting was the Department (P&M) at the University of Applied Sciences, Hamburg, Germany. The Department is a pioneer in the professionalization of health professionals. Furthermore, the Department offers the most diverse range of health degree programs in northern Germany. Young professionals can complete a dual bachelor study program in nursing and midwifery or study interdisciplinary healthcare and management. Experienced professionals from the nursing, therapeutic, and midwifery professions can advance their skills and specialize in master’s programs. Through its application-based research and practice-oriented university teaching, the department makes a valuable contribution to developing solutions for structural and qualitative challenges in healthcare in Germany [28]. Participants and inclusion criteria. We selected three study programs from our department for the survey: Bachelor of Nursing, Bachelor of Interdisciplinary Healthcare and Management (IGM), and Master of Nursing (n = 135). Students were invited to participate in the survey if they met our following inclusion criteria: (i) study in the comprised degree program, (ii) students aged 18 and older, (iii) active and enrolled in the winter semesters in 2020/21 and 2021/22 (survey time: first academic year). As this was an exploratory cross-sectional survey of a selected survey cohort, no power analyses or sample size calculations were carried out in advance.

## 3. Measures

Survey instrument. The questionnaire contains standardized instruments for measuring health indicators (see variable description below), self-developed questions, and socio-demographic information. The questionnaire items were compiled by a co-author (PK). A pretest was conducted with colleagues from the Department. The main purpose of the pretest for the online questionnaire was to check the clarity of the questions and to ensure that the online link would allow participants to answer the questionnaire easily from mobile devices.

Socio-demographic variables. The questionnaire included seven socio-demographic items: age (in years), gender (f, m, d), degree program, initial semester, country of birth, nationality, and highest level of German schooling/vocational education.

### 3.1. Health Status Variables

Four health indicators were used to assess subjective health status.

Subjective health. Health status was measured by the subjective health-related assessment (1 = excellent, 2 = very good, 3 = good, 4 = not so good, 5 = poor) [29].

Body mass index. Self-reported body height and body weight were used to calculate body mass index (BMI). BMI was calculated as the ratio of body weight to the square of the body height (kg/m^2^) and classified as underweight (BMI < 18.5 kg/m^2^), normal (BMI between 18.5 kg/m^2^ and under 25 kg/m^2^), overweight (BMI between 25 kg/m^2^ and under 30 kg/m^2^), or obese (BMI > 30 kg/m^2^) [30]. A categorial variable was formed for analysis.

Health conditions. Health conditions were assessed using the short version of the Work Ability Index [31], which gathers information on current diseases or diseases in the last 12 months. This short version contains a list of nine items to be answered on three response options: ‘no disease’, ‘yes, own diagnosis’, ‘yes, diagnoses from doctor’. According to the research aim, a health condition was considered for subjective health status if at least one response was ‘yes, diagnosis from doctor’. As a result, a dichotomous variable was formed (0 = no-medical diagnosis; 1 = medical diagnosis) to differentiate between students with and without formal health conditions.

Mental well-being. Mental well-being was assessed using the WHO-5 Well-Being Index [32], which measures self-reported mental well-being over the past 2 weeks. This index is a short questionnaire containing 5 simple and non-invasive questions. The Well-Being Index was evaluated according to the recommended procedures. Thus, the total score for assessing mental well-being is calculated by simply summing the 5 item values. This index provides a total score between 0 and 25. A score of 0 to 13 points represents low mental well-being and a score of 14 to 25 points to high mental well-being over the past two weeks. According to Brähler et al. (2007) [32], cases with missing values were excluded from the analyses. For the present analysis, a dichotomous variable was used (1 = high mental well-being; 2 = low mental well-being) to differentiate between students with high mental well-being and students with low mental well-being.

### 3.2. Health Behavior Variables

For assessed frequencies of health-related behaviors, four dimensions were used:

Physical activity. We investigated the frequency of physical activity by asking, “How often are you physically active?” [33], p. 766. Participants could report how many hours per week they are physically active in general. The physical activity questions contained five response options: “no-physical activity”, “less than 1 h per week”, “regular, 1–2 h per week”, “regular, 2–4 h per week”, “regular, more than 4 h per week”.

Smoking habits. To measure smoking habits, a question asking, “Are you currently smoking (e.g., cigarettes, cigars, cigarillos)?” was used [34]. The participants had four response options: “Yes, daily”, “Yes, occasionally”, “No, I have smoked in the past”, “No, I have never smoked”. Smoking habits were included in health-related behavior aspects if the response was “Yes, daily”.

Alcohol use. Alcohol use was assessed using the AUDIT-C [35], a brief screening test for risky alcohol use. The analysis of alcohol use was completed according to the procedure of Bush et al. (1998) [35]. The analysis procedure provides for the formation of a sum score. The sum score reaches a maximum of 12 points. A score of 5 (for men) or more and a score of 4 (for women) or more indicates risky alcohol use. For the analysis, a dichotomous variable was used (1 = non-risky alcohol use, 2 = risky alcohol use).

Eating habits. Eating habits were assessed using a short qualitative food frequency list [36]. Participants self-reported their food intake in general. Thus, six frequency categories were possible (6 = almost daily, 5 = several times per week, 4 = about once a week, 3 = several times per month, 2 = once a month or less, 1 = never). The analysis of eating behavior was based on the framework proposed by Winkler and Döhring (1998) [36]. By querying the six frequencies of consumption of 15 food groups (fast food was excluded), the formation of a nutrition index is possible. The points are summed. After that, points were categorized into three groups: 0 to 12 points for “unhealthy eating habit” group, 13 to 15 points for “normal eating habit” group, and 16 to 30 points for “healthy eating habit” group. This resulted in a categorical variable for the analysis.

### 3.3. Health Literacy Level

The level of health literacy was assessed using the HLS-EU-Q16 [37]. This short version is a validated German Health Literacy Questionnaire, which covers 16 items in the fields of healthcare, disease prevention, and health promotion. The 16 items were assessed on a 4-point Likert scale (1 = very difficult, 2 = difficult, 3 = easy, and 4 = very easy) in dealing with and understanding health-related information. The data were prepared and analyzed according to the procedure used by Röthlin et al. (2013) [37]. Therefore, the four-level response categories were binarized (0 = very difficult/difficult and 1 = easy/very easy) to calculate a total score from 0 to 16 points. Afterward, the scores were categorized into three health literacy levels: 13 to 16 points for “sufficient health literacy”, 9 to 12 points for “problematic health literacy”, and 0 to 8 points for “inadequate health literacy”. Cases with more than two missing items were excluded from the analysis. The categorized level groups resulted in a categorical variable for the present analysis.

## 4. Data Analysis

Survey participants were described in terms of absolute and relative frequencies, means, and standard deviations. Variables of health status, health-related behaviors, and health literacy were described in terms of absolute and (valid) relative frequencies. To examine differences between the student groups, both the Pearson chi-square test for categorical variables and the Fisher exact test were used if the expected cell frequencies were less than five. In addition, the t-test for independent samples and the Mann-Whitney U-test for non-normally distributed data were used [38]. A *p*-value < 0.05 was considered significant. Statistical analyses were performed using IBM SPSS software (version 29).

## 5. Results

### 5.1. Demographic Characteristics of the Participants

The socio-demographic characteristics of the participants are presented in Table 1. In this study, a total of *n* = 98 health professional students participated in the online survey (response rate 72.6%). The mean age of the students was 26.5 years. It should be noted that the students in the Interdisciplinary Health and Nursing Management (IGM)/Master’s Nursing Bachelor’s degree program were on average eight years older (30.6 years) than their fellow students in the Nursing Bachelor’s degree program (22.8 years) (*p* = 0.001). A higher proportion of participants were female (*n* = 76, 77.6%). Most of the students were enrolled and active in a Bachelor of Nursing program (*n* = 51, 52.0%), followed by Bachelor of IGM (*n* = 34, 34.7%) and Master of Nursing (*n* = 13, 13.3%) programs. A total of 7.2% of students were not German citizens. The IGM and Master’s students reported that they had completed vocational training or a Bachelor’s degree in a healthcare profession.

### 5.2. Health Status, Health Behavior, and Health Literacy Level of the Participants

Results of the first and second questions: How do health professional students assess their health status, health-related behavior, and health literacy level at the beginning of their studies? Are there differences between students in terms of their health status, health-related behavior, and health literacy level?

The health situation of the participants is presented in Table 2. The general result showed that 7.3% rated their health as *excellent*, while the majority (80.3%) rated their health as *very good* or *good*. In terms of body mass index, 70.8% had a normal BMI, while 24% were considered overweight or obese. Over 62.1% of the participants had at least one medically diagnosed condition at the start of the study. In terms of mental well-being, more than half of the participants (59.0%) reported a high level of mental well-being.

In the area of health behavior, around 40.6% of the students stated that they were physically inactive. Daily smoking consumption was 20%, while 24% reported risky alcohol intake behavior. More than half of the respondents (63%) reported a healthy diet. In terms of health literacy level, around 55% of students rated their health literacy as sufficient, while 45.3% rated their health literacy as low.

Considering the study groups, bachelor and IGM/master students showed different frequencies in the health outcomes. Bachelor students were significantly more likely to be smokers (21.6% vs. 17.8%, *p* = 0.005), while IGM/master students showed a higher prevalence of regular physical activity (73.3% vs. 47%, *p* = 0.007). In terms of health status, body mass index, medical conditions, mental well-being, alcohol use, and eating habits, there were also differences in frequency, but these were not significant between the two groups.

In terms of health literacy level, participants showed no significant differences. Interestingly, the analysis of the health literacy score (0 to 16 points) exposed a significant variance between the groups. The mean score was 13.5 (2.3) for students in the IGM/master’s program and 12.3 (3.0) for bachelor students (*p* = 0.029). Furthermore, there were no significant differences between students who had completed training in a health profession (*p* = 0.879). 

### 5.3. Health Variables and Health Literacy Level

Results of the third question: Are there differences in the prevalence of health and health-related behavior between students with sufficient and low health literacy?

Table 3 shows the variables collected on health status and health behavior, grouped according to the level of health literacy. It was found that students with low health literacy had a higher prevalence of obesity (26.2% vs. 19.6%), medical conditions (65.2% vs. 59.6%), and low mental well-being (53.3% vs. 30.8%, *p* = 0.036) compared to their peers with sufficient health literacy. About health-related behavior, it was found that students with low health literacy are more often physically inactive (65.1% vs. 61.5%), have risky alcohol consumption (25.6% vs. 23.1%), and engage in unhealthy eating habits (48.8% vs. 26.5%, *p* = 0.032). The prevalence of smoking was 2.6 percentage points higher among students with sufficient health literacy. In terms of subjective health status, there are no substantial differences in frequency according to health literacy level.

## 6. Discussion

Summary of key results. Our survey aimed to explore and describe the health status, health-related behavior, and health literacy level of first-year health professional students. Additionally, we intended to explore potential differences between the two groups of students. While most surveyed students rated their health as *good* or *very good*, some faced weight issues, and a significant number reported health conditions at the beginning of their studies. Many students reported good mental health, and most considered their health literacy to be sufficient. Significant frequency differences were observed in terms of smoking and physical activity between the two groups, though the exact reasons for these differences were not explored in detail. The health literacy score varied slightly between the groups, with IGM/master students showing a higher average score. To ensure a comprehensive perspective within the framework of the health project on “Self-Care”, the SAR model should be employed. This approach not only facilitates an in-depth analysis of internal requirements but also considers external demands, particularly in the context of practical placements in the healthcare setting. Incorporating the SAR model can contribute to ensuring a holistic approach that encompasses all relevant aspects of self-care and external requirements. A suitable study-related module, such as “Health Promotion and Prevention,” could, within the SAR model framework, provide the appropriate structure for a one-semester health project.

Interpretation of results considering other research/evidence. Demographic characteristics. In our study cohort, female students were significantly overrepresented, which is consistent with other studies that have also found a significantly higher proportion of female students compared to their male peers [39,40]. However, it is worth noting that this overrepresentation did not occur in the first nationwide student survey, which reported an almost even gender distribution [6]. The gender composition of the health professions in Germany, which is 75% female, contextualizes this gender discrepancy [41]. Furthermore, there was a notable age difference between our students. Nursing students were significantly younger than the advanced students. This could be because nursing students are usually at the beginning of their careers, while older students may have already acquired their first professional qualifications [42]. The average age of students in the nationwide survey was 26.4 years [6], which highlights the comparatively youthful profile of our surveyed nursing students. It is crucial to consider demographic characteristics such as gender and age, as they play an important role in health outcomes and resources, e.g., health literacy due to their persistence [43,44]. These characteristics may lead to differences in the prevalence of health status and behaviors [45], which could influence the results and interpretations of our study. It is worth noting that male study participants were under-represented in our study, although current trends in the German health system show an increase in the proportion of young men from 19% to 25% [46]. Therefore, the study of male students’ health and their access to health promotion is becoming increasingly important [47]. In view of this, it is crucial to keep gender-specific aspects in health promotion activities in the university and workplace environment. Health status characteristics. A significant percentage of students (82%) in Grützmacher et al.’s nationwide cross-sectional survey also rated their health status as good or very good [6]. In the GEDA survey, as many as 80% of young adults (18–44 years) rated their health status as good or very good [45]. Interestingly, students in our cohort rated their health status more positively compared to a reference group of the same age in the general population [45].

In a cross-sectional study, conducted at a German university, it was observed that young nursing students experience a notable decline in their mental quality of life, potentially linked to the challenges associated with the transition to university life [40]. Overall, it is difficult to explain this result; possibly it could be related to the fact that starting a degree program is a particularly challenging new life stage for young people, which increases subjective stress levels [1,2]. Thus, it appears that nursing students may require tailored health promotion strategies in the early stages of their studies. In our study cohort, the prevalence of weight disorders was 24%. Recent data show that 26.2% of 18-to-29-year-old people in the German population self-report being overweight/obese [48]. The prevalence we found in our study is slightly lower than this reported value. However, the prevalence of overweight/obesity was similar among our nursing students. The impact of obesity is multifaceted: it is a significant risk factor for non-communicable diseases such as type 2 diabetes, cardiovascular disease, certain cancers, and musculoskeletal disorders [30,48]. Furthermore, there is an association between obesity and an increased risk of early death [49,50]. Thus, eating habits play a critical role in subjective health status and performance. Within the Prevention Guide, this topic holds a central position and is supported by primary prevention initiatives of German health insurance companies. Students were defined as a specific target group for health promotion [9]. As a result, there is a need for evidence-based strategies to address and prevent overweight and obesity among our future health professionals. Students are generally considered to be a relatively healthy population group [51]. More than half of our study participants reported at least one medically diagnosed condition. The most common conditions were disorders of the skin (22.3%, *n* = 21), the musculoskeletal system (20%, *n* = 19), and metabolic diseases (12%, *n* = 11). These findings seem to be consistent with other research results, which found that young students most commonly complain of limb pain and shoulder, back, or neck pain [6,52,53]. In the German population, musculoskeletal disorders are also the most common diseases. Their manifestations and causes vary, and some diseases can also be caused by work [24,54]. This could possibly apply to our survey participants and account for the worrying result because they are already involved in the occupational practice. The high frequency of reported skin diseases may also be related to occupational activity. According to the German Social Accident Insurance, hand eczema is the most frequently reported disease among employed persons. This particularly affects employees in the healthcare sector. For the prevention of work-related skin diseases, accident insurance provides appropriate offers [55]. Despite the restrictions related to the COVID-19 pandemic, it is notable that over half of our students rated their mental well-being as high. This finding is important both in the context of research on student mental health and in the larger context of health professionals in Germany [24,56]. For instance, Giesselbach et al. (2023) emphasized that 68% of students exhibited signs of depressive syndrome, while surprisingly, only 32% of their study participants rated their mental well-being as good [57]. Further research results indicate that female students are more prone to burnout symptoms such as exhaustion [6]. Another study focused on non-health-related aspects such as time pressure and academic demands, which may potentially influence students’ subjectively perceived stress levels significantly. These findings are consistent with the observation that bachelor’s students rate their subjective stress experience as particularly pronounced [7]. In addition, the analysis of routine data from Germany’s largest health insurance company showed that the proportion of students with depressive symptoms who were prescribed antidepressants was higher than the proportion of no-student peers [58]. These findings suggest that despite a significant number of students with stable mental well-being at baseline, a significant group continues to cope with mental health issues, which both our findings and the literature underscore [7,40,59]. Women in this professional field often face challenges balancing work and family life, which is especially important due to the high proportion of women in this sector [17,41]. As a result, consequences include a low job retention rate, increasing absenteeism, and significant turnover [17,19]. More recent demands in professional practice highlight the importance of mental health and the promotion of resilience [60,61]. Therefore, it is becoming increasingly urgent to pay more attention to this topic in academic institutions and workplace settings. In this context, we argue that proactively managing burnout symptoms during studies and strengthening personal resources and skills are of great importance for future careers. Health behavior characteristics. In summary, our survey revealed noteworthy insights into health-related behaviors among the survey participants. Our survey showed that only one-third of the participants met the WHO activity recommendations [62]. Our students in Bachelor of Nursing programs were particularly affected by this issue. Study-related tasks are largely spent sitting. As a result, promoting physical activity is an important issue during the study period [63]. Physical activity and regular exercise do improve physical and mental health or sleep outcomes and prevent non-communicable diseases, such as cardiovascular diseases [62,64]. Consequently, this research topic is of increasing relevance in the university setting, and students are an important target group for physical activity promotion measures. In addition, our data showed that 20% of the participants smoke daily. Compared to the nationwide student survey, smoking prevalence was 18.5% [6]. In our study group, smoking prevalence is slightly higher. Smoking represents the greatest health risk and is the main cause of premature death [34]. Thus, the German Cancer Research Center emphasized the importance of not smoking by publishing a comprehensive strategy paper for a tobacco-free Germany initiative [65]. Our data highlight a crucial need for effective smoking cessation interventions for our students. Furthermore, we were able to identify a prevalence of 24% for risky alcohol consumption. A comparison with the general population shows something similar: the proportion of people with risky alcohol consumption is 24%, especially among 18–29-year-olds [66]. Germany is considered a high-consumption country by international standards [67]. Promoting responsible consumption is, therefore, a challenging task. In this context, target group-specific interventions have been shown to be an effective method to support students in developing responsible alcohol consumption [68]. In this respect, we see a need for intervention in our setting. In terms of their eating habits, most students try to eat healthy. Nevertheless, the time they spend at university can affect their efforts to maintain balanced eating behaviors [53]. Healthy nutrition, coupled with an active lifestyle, plays a central role in the prevention of non-communicable diseases [69]. Given the prominent importance of eating habits for personal health, as well as increasingly for planetary health, this topic is of public health relevance [9]. It should be adequately addressed in health-promoting interventions. Despite most students paying attention to their nutrition, there exists a proportion that practices unhealthy eating behaviors. In our opinion, this calls for targeted approaches to intervention for this specific group of students, also in light of the future field of professional action. In summary, health-related behaviors are of particular importance to overall health. Physical inactivity, smoking, risky alcohol use, inadequate diet, and obesity are major contributors to non-communicable chronic diseases [70,71]. Furthermore, our students are future health professionals who take on an important role-model function for the health of their patients. Therefore, the promotion of one’s own health is of particular importance already during one’s studies. Health literacy level. More than half of our students rated their health literacy as sufficient. This result contrasts with previous studies, most of which found low health literacy both in the general German population [44,72] and among health professional students [39,73]. Regarding socio-demographic variables, our findings are consistent with other health professional student surveys that found no significant differences between health literacy level and gender or education [39,73]. Moreover, our surveyed students with low health literacy were more vulnerable to specific health issues and critical health-related behavior. This finding seems to be in line with some studies that have found associations between health literacy levels and various health indicators [44,72,74,75]. Our results suggest that some students already have health literacy that enables them to understand, evaluate, and use health-related information. In summary, health literacy is an important field of action for health promotion to ensure that all students enter professional life with good health literacy.

## 7. Limitations

Our survey has some limitations that need to be considered when interpreting the results. Since we collected data at a specific point in time, our survey does not allow us to draw conclusions about cause–effect relationships but only to identify differences between variables. It is important to emphasize that our study was designed as an exploratory analysis, especially in light of the specifically selected target group. The sample is not representative and, therefore, no generalizations should be made from the study participants to the target population. A more comprehensive analysis of the health situation would require representative data, which are important for future research. The disproportionate representation of female students in our cohort could possibly reflect gender distribution patterns in the health sector [41] and lead to variations in health characteristics that may not necessarily be transferable to other student groups, especially when the proportion of women varies in different fields of study. Despite the limited representativeness, our sample size was sufficient to gain important insights into the health situation of our freshmen. It is important to note that the data collection was based on self-reporting, which led to potential bias. The survey took place during the COVID-19 pandemic, which may have influenced students’ health perceptions and possibly led to under- or overestimations of various health aspects. In addition, the reliability of the results may have been affected by the possible exclusion of students with existing health problems who did not participate in the study.

## 8. Conclusions and Practical Implications

Early health promotion in the university setting plays a significant role for the examined student cohort. More than half of the participants reported health conditions at the beginning of their studies, and unhealthy behaviors such as physical inactivity, smoking, risky alcohol use, and unhealthy eating habits were evident. Regarding health literacy, it is noteworthy that half of the students from both study groups have low health literacy. In summary, students with low health literacy seem to be more vulnerable to certain health issues and unfavorable behaviors. These findings could serve as a basis for targeted interventions to improve health literacy. The promotion of health literacy is not only relevant in terms of personal health and health behavior but also crucial in the context of the demanding professional practice in healthcare. Integrating health literacy education into the training of health professionals can enhance their ability to navigate complex healthcare environments and communicate effectively with diverse patient populations. The cross-sectional survey expands the knowledge of the health situation of this student group in Germany and addresses a research gap. The findings are not only scientifically relevant but also offer practical insights for educational institutions and healthcare providers to develop targeted health promotion strategies in the workplace setting. We also recommend further research. For example, qualitative research approaches can further explore and deepen students’ subjective needs and the importance of their own health. This proposal aims to broaden our understanding of the complex health dynamics within this student population and support future health promotion initiatives.

## Figures and Tables

**Table 1:** Socio-demographic characteristics of the participants (*n* = 98).

	First-Year Students (*n* = 98)
	*n* (Valid %)
**Socio-Demographic Variables**	
**Age (in years)**	
Mean (SD) (range)	26.5 (7.1) (19–62)
Mean (SD) (range) (Bachelor of Nursing)	22.8 (3.4) (19–37)
Mean (SD) (range) (Bachelor of Interdisciplinary Healthcare and Management/Master of Nursing)	30.6 (8.6) (20–62)
**Gender**	
Female	76 (77.6)
Male	22 (22.4)
Diverse	0
**Courses of study at the department**	
Bachelor of Nursing	51 (52.0)
Bachelor of Interdisciplinary Healthcare and Management (IGM)	34 (34.7)
Master of Nursing	13 (13.3)
**Initial Semester**	
Winter semester 2020/21	76 (77.6)
Winter semester 2021/22	22 (22.4)
**Country of birth**	
Germany	85 (88.5)
Other background	11 (11.5)
missing	2
**Nationality**	
Germany	90 (92.8)
Other background	7 (7.2)
missing	1
**Highest level of schooling**	
German High School Diploma	72 (73.5)
Technical Diploma	21 (21.4)
Secondary School	5 (5.1)
Students in the IGM or Master of Nursing program already have training or a bachelor’s degree in a health professional program.	47 (100)

**Table 2:** Group comparison of health status/health behavior variables and health literacy level.

	Students Total (*n* = 98)	Bachelor of Nursing (*n* = 51)	Bachelor of IGM/ Master of Nursing (*n* = 47)	*p* *
	*n* (Valid %)	*n* (Valid %)	*n* (Valid %)	
**Health status variables.**				
**Subjective health.**				0.204
excellent	7 (7.3)	6 (11.8)	1 (2.2)	
very good/good	77 (80.3)	37 (72.6)	40 (88.9)	
not so good	12 (12.5)	8 (15.7)	4 (8.9)	
poor	0 (0.0)	0 (0.0)	0 (0.0)	
missing	2	0	2	
**Body mass index.**				0.451
underweight	5 (5.2)	2 (4.1)	3 (6.4)	
normal	68 (70.8)	34 (69.4)	34 (72.3)	
overweight/obese	23 (24.0)	13 (26.5)	10 (21.3)	
missing	2	2	0	
**Medical conditions.**				0.832
no-medical diagnosis	36 (37.9)	18 (36.0)	18 (40.0)	
medical diagnosis	59 (62.1)	32 (64.0)	27 (60.0)	
missing	3	1	2	
**Mental well-being.**				0.838
high	56 (58.9)	30 (60.0)	26.0 (57.8)	
low	39 (41.1)	20 (40.0)	19.0 (42.2)	
missing	3	1	2	
**Health behavior variables.**				
**Physical activity.**				0.007 (Cramer’s V = 0.38)
no-physical activity/ less than 1 h/week	39 (40.6)	27 (53.0)	12 (26.7)	
regular, 1–2 h/week	22 (22.9)	12 (23.5)	10 (22.2)	
regular, 2–4 h/week/ regular, more than 4 h/week	35 (36.4)	12 (23.5)	23 (51.1)	
missing	2	0	2	
**Smoking habits.**				0.005 (Cramer’s V = 0.36)
Non-smoker	77 (80.2)	40 (78.4)	37 (82.2)	
Smoker	19 (19.8)	11 (21.6)	8 (17.8)	
missing	2	0	2	
**Alcohol use.**				0.917
non-risky alcohol use	73 (76.0)	39 (76.5)	34 (75.6)	
risky alcohol use	23 (24.0)	12 (23.5)	11 (24.4)	
missing	2	0	2	
**Eating habits.**				0.797
healthy/normal	58 (63.0)	32 (65.3)	26 (60.5)	
unhealthy	34 (37.0)	17 (34.7)	17 (39.5)	
missing	6	2	4	
**Health literacy level.**				0.202
sufficient	52 (54.7)	25 (50.0)	27 (60.0)	
problematic/inadequate	43 (45.3)	25 (50.0)	18 (40.0)	
missing	3	1	2	

* Pearson’s chi^2^ test/Fisher’s exact test.

**Table 3:** Health variables grouped according to health literacy level.

Collected Variables		Level of Health Literacy		
Health Variables	Expressions	Sufficient	Problematic/Inadequate	p *
		Absolute Frequency (%)		
**Health status**	excellent	6 (11.5)	1 (2.3)	0.279
	very good/good	40 (77.0)	36 (83.7)	
	not so good	6 (11.5)	6 (14.0)	
**Body mass index**	overweight/ obese	10 (19.6)	11 (26.2)	0.103
**Health condition**	medical diagnosed	31 (59.6)	28 (65.2)	0.673
**Mental well-being**	high	36 (69.2)	20 (46.5)	0.036 (Cramer’s V = 0.23)
	low	16 (30.8)	23 (53.5)	
**Physical activity**	>2 h/week	20 (38.5)	15 (34.9)	0.831
	<2 h/week	32 (61.5)	28 (65.1)	
**Smoking habit**	daily	11 (21.2)	8 (18.6)	0.876
	non	41 (78.8)	35 (81.4)	
**Alcohol use**	risky	12 (23.1)	11 (25.6)	0.813
	non-risky	40 (76.9)	32 (74.4)	
**Eating habit**	healthy/normal	36 (73.5)	22 (51.2)	0.032 (Cramer’s V = 0.23)
	unhealthy	13 (26.5)	21 (48.8)	

* Pearson’s chi^2^ test/Fisher’s exact test.

## Data Availability

Data are available on request due to restrictions, e.g., privacy or ethical. The data presented in this study are available on request from the corresponding author. The data are not publicly available due to the fact that this was not subject to the informed consent.
